# Optimized optogenetic anti-CRISPR for endogenous gene regulation in *Drosophila*

**DOI:** 10.1093/nar/gkag244

**Published:** 2026-05-05

**Authors:** Clarissien Ramongolalaina, José C Pastor-Pareja, Emily Zhang, Yichang Jia

**Affiliations:** Aging and Regeneration Center, School of Basic Medical Sciences, Tsinghua Medicine, Medical Science Building, Room D204, Tsinghua University, Beijing 100084, China; The State Key Laboratory for Complex, Severe, and Rare Diseases, Beijing 100084, China; School of Life Science, Tsinghua University, Beijing 100084, China; IDG/McGovern Institute for Brain Research at Tsinghua, Beijing 100084, China; Tsinghua–Peking Joint Center for Life Sciences, Beijing 100084, China; School of Life Science, Tsinghua University, Beijing 100084, China; Institute of Neurosciences, Consejo Superior de Investigaciones Científicas-Universidad Miguel Hernández, San Juan de Alicante 03550, Spain; Tsinghua International School (THIS), Tsinghua University, Beijing 100084, China; Aging and Regeneration Center, School of Basic Medical Sciences, Tsinghua Medicine, Medical Science Building, Room D204, Tsinghua University, Beijing 100084, China; The State Key Laboratory for Complex, Severe, and Rare Diseases, Beijing 100084, China; School of Life Science, Tsinghua University, Beijing 100084, China; IDG/McGovern Institute for Brain Research at Tsinghua, Beijing 100084, China; Tsinghua–Peking Joint Center for Life Sciences, Beijing 100084, China; SXMU–Tsinghua Collaborative Center for Frontier Medicine, Shanxi Medical University, Taiyuan 030001, China; Tsinghua Laboratory of Brain and Intelligence, Tsinghua University, Beijing 100084, China

## Abstract

Optogenetic tools—light-responsive proteins that enable to regulate specific cellular activities, study biological processes, and develop new therapies—are attractive approaches for achieving endogenous gene regulation under minimally invasive conditions. Our first step in constructing an optogenetic system to regulate endogenous *Drosophila* gene expression was to identify inhibitory anti-CRISPR (Acr) proteins that block CRISPRa-mediated activation. Next, we inserted optogenetic protein LOV2 into these Acrs, tested for their ability to optogenetically modulate endogenous gene upregulation through the CRISPRa-based flySAM system in *Drosophila*, and found that the photoswitchability of these prototypes was weak. We therefore engineered an optimized Acr–LOV2 fusion module by refining length of intrinsically disordered and ordered regions (IDR and IOR) of Acrs. This optimization yielded a variant with significantly greater sensitivity to blue-light-induced endogenous gene upregulation than the prototypes, leading to new *in vivo* discoveries. In addition, this work provides insights for *in vivo* functional characterization of the IDR and the IOR of these small-sized proteins. Together, these findings establish a robust optogenetic toolbox for precise, light-controlled endogenous gene regulation in *Drosophila*.

## Graphical Abstract

**Graphical Abstract:**
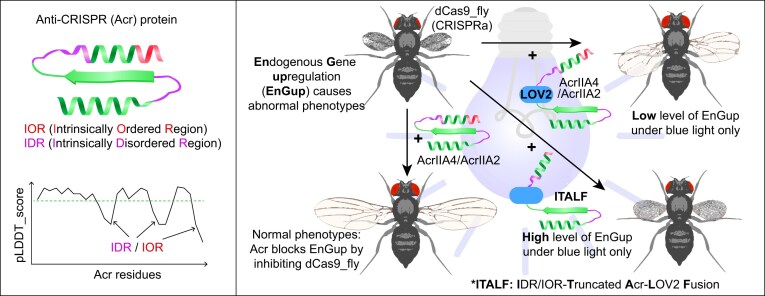

## Introduction

Optogenetics, the integration of optics and genetics, can be defined as an approach to control the function of an “opsin” protein using light. Originally a cornerstone of neuroscience, it has also been harnessed by the biotechnology field to spatiotemporally modulate gene expression, protein interactions, and countless biological reactions not exclusively for research purposes, but for therapeutic applications as well [1]. Its implementation to regulate endogenous gene expression *in vivo* is promising; this owes to light features such as sharpness, spatial resolution [2], and spectral multiplexing [3]. In model organisms such as *Drosophila*, existing systems enable efficient optogenetic control of genes or proteins are expressed from transgenes [4–6]. In contrast, endogenous gene regulation in *Drosophila* is currently achievable via CRISPRa [7]—hereafter named dCas9_fly—and CRISPRi [8], but these systems lack inherent light-switchability.

In engineering light-switchable tools, understanding the detailed structure of the protein of interest, more distinctively its intrinsically disordered regions (IDRs), is critical. IDRs are ideal switch sites for insertion of an opsin [9], although IDR-mediated interactions often differ between *in vitro* and *in vivo* systems, as demonstrated in yeast and mouse models [10–15]. IDR—a peptide that has multiple conformations, without 3D structure [16]—is generally a crucial part of any given proteome, and is associated with many functions, including cell signaling and protein interactions [17], and the formation of membraneless organelles [18]. Accordingly, several approaches have been developed for the prediction of IDRs; among those proven to be accurate is the AlphaFold2-defined per-residue local distance difference test (pLDDT) scores of protein models [19–21]. Intriguingly, the theoretical computation of pLDDT scores leads to the identification of 3D-folded IDRs [21] that would not be otherwise classified as IDRs and have never been experimentally characterized. New web servers such as MobiDB [22] have been continuously emerging for the prediction and characterization of IDRs; however, they do not provide structural details of small-sized proteins such as anti-CRISPR (Acr) proteins.

Acr bacteriophage proteins act as natural antagonists of CRISPR–Cas systems. Amongst the 122 nonhomologous Acr proteins discovered to date, and classified into four types, the type II families can inhibit DNA cleavage by CRISPR–Cas9 [23]. In doing so, Acr proteins, controlled with opsins, can serve as proper tools to optogenetically modulate CRISPR–Cas9 activities. The LOV2 photoreceptor from *Avena sativa* (oat) is the most used opsin in research. In the presence of the chromophore flavin mononucleotide, a cofactor that exists in almost all species, it can be optimally and reversibly activated by 450 nm blue light [24]. In the last few years, type II Acr proteins inserted with LOV2 have shown CRISPR–Cas9 light-switchability *in vitro* human cell cultures [25, 26], in *Escherichia coli* [27] and mosquitoes [28].

Despite the promise of optogenetic CRISPR for endogenous gene regulation in *Drosophila*, several key challenges remain: Can an Acr protein be efficiently functional in *Drosophila*? If so, how to engineer a photoswitchable Acr without disrupting its functionality? How would the functional significance of Acr IDRs, the binding affinity of which *in vitro* and *in vivo* may differ, come into play? And how can this system be optimized to achieve light-sensitive phenotypic effects? Addressing these points herein, we identified the Acr proteins that can inhibit dCas9_fly-mediated endogenous gene upregulation (EnGup) in *Drosophila*, analyzed structures of these Acrs to determine their likely optimal insertion sites of LOV2, and generated flies expressing Acr–LOV2 fusion prototypes, which we next optimized mainly by refining the length of structures that we defined as IDRs and intrinsically ordered regions (IORs) in Acrs. Finally, we demonstrated the application of our optimized system in autophagy investigation and phenotypic modeling through manipulation of endogenous gene expression.

## Materials and methods

### Plasmid construction and cloning

All constructs (Supplementary Table S1) were generated with NEBuilder HiFi DNA Assembly Cloning Kit (NEB, #E5520S) or 2× Seamless Cloning Mix (Beyotime, #D7010M). Briefly, DNA sequences synthesized by Xiang Hong (xianghongbio.com) or available plasmids from Addgene were amplified according to 2× Taq Plus Master Mix II protocol (Vazyme, #P213), and used as templates; pValium-ROE10 [29], pScript-Renilla [30], and lentiCRISPR v2 (Addgene, #52961) were linearized, with KOD One™ PCR (Toyobo, #KMM-101), and used as vector backbones for plasmids to be injected in *Drosophila*, expressed transiently and expressed stably in cells, respectively. The fragments and vectors were purified by V-ELITE Gel Mini Purification kit (ZOMANBIO, #ZPV202) and assembled by 2× NEBuilder Hifi Master Mix (NEB, #E2621s) or 2× Seamless Cloning Mix Kit (Beyotime, #D7020S).

### *Drosophila* stocks and fly husbandry

All *Drosophila* stocks were maintained on standard cornmeal/sugar/agar medium [7]. Experiments were conducted at 25°C. *Drosophila melanogaster* stocks used in this study are listed in Supplementary Table S2. Transgenic flies were generated by the injection of plasmids with UAS-driven Acr or Acr–LOV2 fusion proteins and dCas9_fly for *wg* EnGup into *y sc v nanos-integrase; attP40* or *y sc v nanos-integrase;; attP2*.

### Cell culture and stable cell line generation

Human embryonic kidney 293T (HEK293T) cells were cultured in DMEM (Gibco, #C11995500BT) supplemented with 10% (vol/vol) fetal bovine serum (FBS; Gibco, #16000-044) and 1% (vol/vol) penicillin solution (Beyotime, #ST488-1) at 5% CO_2_ and 37°C in a humidified incubator. The cells were polymerase chain reaction (PCR) tested for mycoplasma contamination and passaged every 2–4 days.

For stable cell lines, Cas9 and dCas9-3(VP64) cloned into lentiCRISPR v2 vector plasmids were transfected into HEK293T cells according to the established protocol [31]. Briefly, a mixture of 5 µg of each of these lentiviral plasmids, 2.5 µg of pMD2.G (addgenes, #12259), 3.75 µg of psPAX2 (addgenes, #12260), and 12.5 μl of Lipofectamine 3000 (Invitrogen, #L3000015) in OptiMEM (Life Technologies) was delivered into HEK293T cells in a 60-mm dish (Nest, #706001) at 80%–90% confluency. After 6 h, the culture medium was changed to Dulbecco’s modified Eagle medium (DMEM) with 10% FBS, then added with 4 µg/ml of blasticidin for selection 24 h later. This selection process, followed by cell passaging, was repeated three times before storing the stable cell lines at −80°C in M5 Hiper medium (Mei5Bio, #CS699-01).

### Plasmid co-transfection

For transient expression, stable cell lines were (co-)transfected employing an optimized polyethyleneimine (PEI)-based protocol. Briefly, 5 × 10^4^ cells were seeded into a 12-well plate, cultivated overnight to reach 60%–70% confluency, and co-transfected with 2 μg/well of a plasmid mixture (1:1 sgRNA&Acr/reporter plasmid ratio). The plasmid mixture was diluted in 50 μl OptiMEM, subsequently mixed with 50 μl PEI solution containing 3.2 μl of PEI (1 mg/ml in ddH_2_O; Polysciences, #24765-2) and 46.8 μl OptiMEM, incubated for 15 min at 25°C, and dropwisely added to the cell culture. The culture medium was renewed 6 h after transfection.

### Computational modeling

Although the Acr proteins we used were available from the alphaFold database [32], we performed the modeling of Acr–LOV2 fusion proteins and Acr–dCas complexes using a locally-installed version of the AlphaFold_2.0_multimer.v3 and more recent AlphaFold3 (https://github.com/google-deepmind/alphafold3), along with ColabFold from Github (https://github.com/YoshitakaMo/localcolabfold) and the environmental databases (https://colabfold.mmseqs.com). We compared the top five ranked outputs and selected Rank 1 to prepare the figures, which were rendered, aligned with SpyCas9–sgRNA–AcrIIA4 (PDB 5VW1) as reference, with the UCSF ChimeraX-1.7.1 software (https://www.cgl.ucsf.edu/chimerax/).

### Structural analysis

The pLDDT confidence scores of Acr proteins downloaded from the AlphaFold [32] were retrieved from the atomic coordinates of a protein residue in a pdb file. These coordinates were mapped in 2D using the bio3D library in RStudio.

Electrostatic potential charges and hydrophobicity of surfaces of AcrIIA4 pdb files were rendered by ChimeraX. However, their exact value from each residue was extracted using surface_analysis library (https://github.com/liedllab/surface_analyses). As for the local charge distribution of proteins, unlike the other structural analyses of 3D structured protein, it was extracted from a primary structure. The average charge of each residue was computed with seqinr and bio3d libraries on RStudio.

### Contact map analysis

The Acr–dCas9 complex pdb file was further processed with Jupyter Notebook (version 6.5.4) with nglview library. The distance matrices between Acr and dCas9 chains and between two residues were calculated using biopython and numpy (version==1.23.4) packages, and those with distances below the 8Å threshold were defined as in contacts. Then, *rpy2* and *tzlocal* packages were employed to 2D-map these distances. Then, the contact map was refined by removing the residues of dCas9 with distances above 10Å.

### Luciferase assay

The Luciferase assay was carried out 2–3 days post-transfection. Cells were cultivated in 12-well plates, the cultured media of which were aspirated, washed with 1× Dulbeccos Phosphate-Buffered Saline (DPBS) (Corning, #21-031-CV), and lysed with 100 μl/well of cell lysis buffer (Beyotime, #RG027-1) on ice for 10 min. Then, Firefly Luciferase activities of the cell lysates were measured according to the previously adapted protocol [33]. Briefly, 50 μl of cell lysates were transferred to one well of 96-well white opti-plate (PerkinElmer, #6005290), then mixed with 100 μl Bio-Lite Luciferase Assay Substrate (Vazyme, #DD1202) that had been dissolved with its buffer. The opti-plate, after incubation at room temperature for 10 min, was loaded into GloMax-Multi Microplate Reader (Promega), which was set for steady-Glo to measure the luminescence for 2 s. All results were normalized with a positive control that was loaded in the first column of the opti-plate.

### FACS analysis

Forty-eight hours after transfection, the medium was removed, cells were detached from the each well using trypsin ethylenediaminetetraacetic acid, transferred into 1.5 ml and centrifuged at 300 g for 7 min at 4°C. The supernatant was discarded, and the cells were resuspended in 1× PBS. The fluorescence-activated cell sorting analyses were carried out using FongCyte Series 3-Laser 14-Color (Chellenbio, Beijing Challen Biotechnology Co., Ltd) with the settings: 450 nm laser and a 500/50 filter with a photomultiplier tube set at 275 V. For each sample, 10^5^ cell events were collected.

### Single guide RNA design

Single guide RNAs (sgRNAs) for upregulation of *wg* gene (ID: FBgn0284084) were designed using the *Drosophila* Resource Screening Center Find CRISPRs v2 online tool (www.flyrnai.org/crispr/). Three pairs of sgRNAs, which were additionally screened for potential off targets with cas-OFFinder (http://www.rgenome.net/cas-offinder/) and targeted 300–800 bp upstream of *wg* gene, were selected and synthesized. Each pair of oligo DNA was cloned in flySAM2.0 construct according to the protocol [34]. As for the Luciferase cleavage and EGFP upregulation in HEK293T cells, the sgRNAs were designed manually, and their off-targets were checked with cas-OFFinder. All sgRNAs are listed in Supplementary Table S3.

### Blue light assay

Adult flies were maintained in rearing conditions in the dark: in a box placed in an incubator at 25°C with humidity of 60%–65%. Every parental population was raised in the same conditions and transferred within 24 h into a fresh vial filled with standard fly food [7]. Offspring, 3rd–4th instar larvae or pupae, from these crosses were shortly screened under a microscope, and up to 35 of the correct genotypes were transferred into a 6 cm dish (NEST, #706001), containing 1–2 mm layer of semi-transparent fly food (50% standard fly food media [7], 40% apple juice, 1% agar, 9% water; boiled and cooled down to 70°C before use). The lid-covered and sellotape-sealed dishes were placed either in dark or in a handmade blue light box—a box painstakingly covered with aluminum foil from inside and illuminated with a custom-made 450–460 nm blue light (Xuzhou Ai Jia electronic technology Co.LTD), which was powered by JUNCTEK JDS8080 80 MHz Signal Generator (https://www.junteks.com/). After treatment (for 30–48 h), the offspring were transferred into a fresh vial and returned to the dark conditions.

### Phenotypic analysis

Female adult wings (12–15 per genotype) were dissected, rinsed with ethanol, mounted in a solution of 1:1 acetone to Permount (Thermo Fisher Scientific) on a glass slide, and imaged on Zeiss Axioskop 2 microscope. Representative images were shown for each genotype, but the characteristics (size, length, width, …) of 10 wings were extracted via Python scripts on Spider environment (version 5.4.3) using *OpenCV, Numpy*, and *Skimage* libraries. As for eye phenotypes, CO_2_-anaesthesized 20 female adults were plated on an ~3 mm layer of 0.4% agar (boiled and cooled down to 50°C before use); eye images were taken with a Zeiss Axioskop 2 microscope, and processed with ImageJ (version 2.14.0).

### Confocal imaging

The adipose tissues were dissected from 3rd instar larvae with fine-tip forceps, washed in PBS for 2× 15 min, and mounted on a glass slide with a drop of DAPIVectashield (Vector Laboratories, #H-1200). The mounted tissues were observed under a Nikon AXR NSPARC using the 63×/1.42 NA oil objective or ZEISS LSM780 confocal microscope equipped with an objective 63× oil Plan-Apochromat (NA 1.4). The images were acquired keeping all parameters constant with laser lines at 488 and 640 nm, and processed with NIS-Elements software (Nikon) and Python scripts.

### Viability analysis

Newly hatched adults from each fly line were cultured in an incubator at 25°C with humidity of 60%–65% and a 12-h dark/light cycle. They were transferred every day into new vials with fresh medium to prevent non-age-associated mortality, like accidental trapping on food, and the numbers of dead flies were recorded. These procedures were continued for 75 days or until all flies were dead. The Kaplan–Meier method was used to present survival data, and the log-rank test was used to compare survival distributions.

### Quantitative RT-PCR

Total RNA was isolated from early hatched adults or late pupae; 50 wings per genotype were dissected in Schneider’s medium (Sigma–Aldrich). The Quick-RNA Microprep kit (Zymo Research, #R1050) was used following the manufacturer’s instructions to isolate RNA, then it was incubated with DNase I (Promega, #Z358A) at 37°C for 30 min and treated with the RNA Clean and Concentrator-5 kit (Zymo Research, #R1015). A total of 1 μg of RNA was used as a template for cDNA synthesis using Moloney Murine Leukemia Virus reverse transcriptase (M-MLV RT) (Invitrogen, #28025013). Results were normalized against rp49 expression.

### Statistical analysis

For all experiments, bars indicate means, and individual data points correspond to individual biological replicates. For the *in vitro* experiments, all data represent the mean ± s.d. of three independent experiments (*n* = 3), with three replicates for the Luciferase experiments. For animal experiments, each genotype or treatment consisted of ten female *Drosophila* (*n* = 10) that displayed representative phenotypes. All group comparisons were analysed by one-way ANOVA-test (cut-off *P* < .01) using an R script on Rstudio. No blinding was performed in all experiments.

## Results

### AcrIIA4a and A4b efficiently deactivate dCas9_fly-mediated EnGup in *Drosophila*

First, since no Acr protein has reportedly been used in *Drosophila*, we selected five type II family members that can inhibit CRISPR–Cas9: AcrIIA2 along with AcrIIA4a, AcrIIA4b and AcrIIA6—inhibitors of *Streptococcus* Cas9 [35–37]—from *Listeria monocytogenes, Listeria farberi*, and *Caudoviricetes sp*., respectively; and AcrIIC3—an antagonist of NmCas9 [25]—from *Neisseria meningitidis*, as a negative control (Supplementary Fig. S1A). AcrIIA2, AcrIIA4, and AcrIIA6 inhibit Cas9 activity specifically by competing with the target DNA strand binding [38], by structurally mimicking the PAM and interacting with the RuvC cleavage site [35], and interfering with the PAM binding site [39], respectively. We generated UAS constructs to express each Acr protein under the control of the Gal4–UAS binary expression system and injected them into flies at the *attP40* site on chromosome II. To assess the potential toxicity of the expression of individual Acr proteins *in vivo*, we crossed each generated Acr line with generally expressed Gal4 driver Tub-Gal4, and tissue-specific drivers Mef2-Gal4 (muscle) and BM-40-SPARC-Gal4 (adipose tissue). Although the expression of AcrIIA6 by these three Gal4 drivers caused a significantly shorter lifespan compared to that of controls, the expression of AcrIIA2, AcrIIA4a, AcrIIA4b, or AcrIIC3 produced normal survival rates (Supplementary Fig. S1B–D). In addition, no visible morphological or developmental defects were observed in these animals (Supplementary Fig. S1E), which allowed us to continue our studies *in vivo* by expressing these Acr proteins.

**Figure 1.**
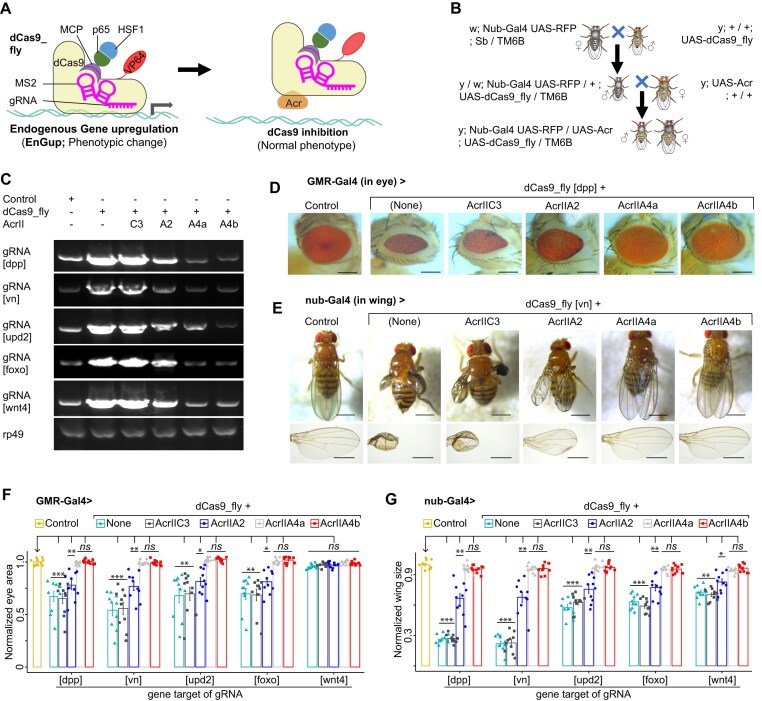
Identification of Acr that effectively inhibits dCas9_flySAM. (**A**) Cartoon representation of dCas9_flySAM for target gene expression in flies. The system is composed of dCas9 fused to transcriptional activation domain p65-HSF1, via MS2-MCP, and to a chimeric activator VP64, directly. Acr protein is hypothetically able to block dCas9_flySAM activity. (**B**) Crossing scheme for coexpressing dCas9_flySAM and Acr with a nonlethal driver, such as Nub-Gal4 in wings. (**C**) Reverse Transcription PCR (RT-PCR) results of fly wings that expressed dCas9_flySAM-targeted a set of genes (*dpp, vn, upd2, foxo*, and *wnt4*) along with each Acr protein. *rp49*, a loading control. Representative images showing that AcrIIA4a&b are effectively able to rescue the phenotypes generated by *in vivo* CRISPRa via inhibition of dCas9 (**D, E**) and corresponding data summaries were shown (**F, G**). Data summaries for eye surfaces (F) and wing sizes (G) of flies coexpressing dCas9_flySAM-targeted genes (*dpp, vn, upd2, foxo*, and *wnt4*) along with each Acr protein. In panels (C)–(G), the Control is a dCas9_fly line without Gal4. In panels (F) and (G), every single value is normalized with the mean of the normal control. Values are represented as means ± s.d.; *n* = 10 female flies. The *P*-values were calculated by ANOVA-test; *ns*, *, ** and *** are, respectively, not significant, <0.01, <1e−3, and <1e−5 compared to the normalized control phenotypes. In panels (D) and (E), scale bars are 500 and 250 μm, respectively. In panels (C)–(G), the “Control” is a dCas9_fly line without Gal4 driver.

Next, we evaluated which Acr proteins could effectively inhibit dCas9 from binding DNA, and thereby prevent dCas9_fly-mediated EnGup in *Drosophila* (Fig. 1A). To that end, we crossed each Acr line flies with dCas9_fly targeted different genes: *dpp, vn, upd2, foxo*, and *wnt4* (Fig. 1B). The transcriptional activation of those endogenous target genes by dCas9_fly was detected as previously described [7]: messenger RNA (mRNA) expressions from dCas9_fly were higher than those from the negative control (Fig. 1C). Of note, although EnGups *in vivo* of the five genes were relatively similar at the mRNA (Fig 1C), those of *dpp* and *vn* produced very consistent and strong phenotypes in eyes and particularly in wings (Fig. 1D–G). More importantly, the inhibition of dCas9_fly-mediated EnGup *in vivo* was evidenced at the mRNA level, since upregulation of the five endogenous target genes was inhibited by expression of AcrIIA2, AcrIIA4a, or AcrIIA4b, but not AcrIIC3 (Fig. 1C). Correspondingly, supported by phenotypic effects, AcrIIA4a and A4b consistently demonstrated stronger inhibition than AcrIIA2: the eye and wing defects induced by dCas9_fly activation were largely reversed by coexpression of AcrIIA4a or A4b, but partially by AcrIIA2 (Fig. 1D–G and Supplementary Fig. S2A–E).

Additionally, EnGups in adipose tissues, which are usually fatal, can be rescued by the expression of AcrIIA2 and, more reliably, AcrIIA4 (Supplementary Fig. S3A and B). Of note, even the ubiquitous expression of dCas9 alone without any sgRNA at a high level is 100% lethal [7]. The coexpression AcrIIA4/A2-dCas9, however, might disrupt some immune system pathways as it increased the mRNA level of *Prophenoloxidase 2* (*PPO2*), a protein of the defense system in *Drosophila* [40, 41] (Supplementary Fig. S3C and D). Although the efficiency of AcrIIA2 in inhibiting dCas9_fly was lower than of AcrIIA4a and A4b, it could increase if the temperature condition is 18°C or lower, or probably if the AcrIIA2 titration is adjusted at 25°C (Supplementary Fig. S3E and F). AcrIIA2 barely inhibits Cas9 in mammalian cells and at 37°C, and reduces only up to 25% of Cas9 activities in *E. coli* as previously demonstrated [42, 43]. Together, these results demonstrate that AcrIIA4a and A4b effectively suppress dCas9_fly-mediated EnGup, and AcrIIA2 also could potentially do so in *Drosophila*.

### The residues adjacent to IDR are predicted to be the favorable sites for opsin LOV2 insertion

**Figure 2.**
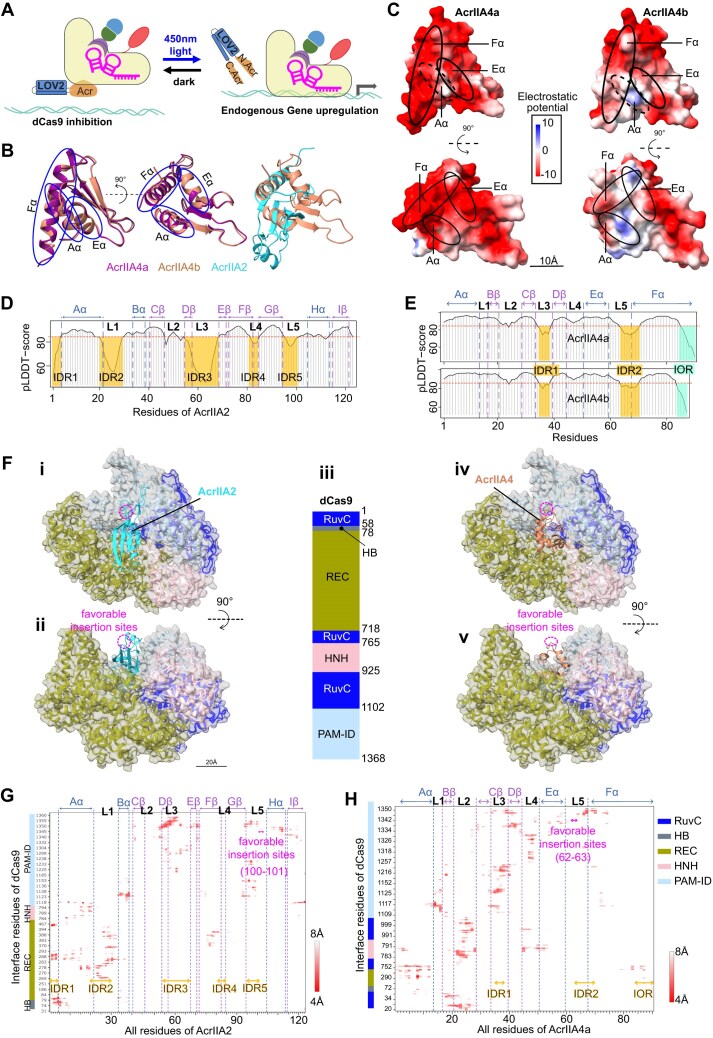
Structural analyses of Acr proteins. (**A**) Schematic of the system that we intended to build in *Drosophila* to enable gene overexpression only under the light illumination. (**B**) Side views of ribbon representation of AcrIIA4a, AcrIIA4b (homology PDB ID 5VW1) and AcrIIA2 (homology PDB ID 6MCB), colored in warm purple, light caramel, and bright cyan, respectively, captioning the structural similarities of the two AcrIIA4(s). The first view (left) is rotated to 90° angle in the second (middle) along the vertical axis (dashed line). The alpha-chains of AcrIIA4(s) (Aα, Eα, Fα) are highlighted. (**C**) Top (left) and side (right) views of electrostatic potentials of the molecular surfaces of AcrIIA4a (upper) and AcrIIA4b (bottom). The predicted local distance difference test (pLDDT) scores of AcrIIA2 (**D**) and AcrIIA4a&b (**E**). Their IDRs and IORs below the threshold (pLDDT_score = 85, in red dashed-line) are labeled and spotlighted, respectively, in semitransparent orange and turquoise. (F, G) Predicted interaction of AcrIIA2 and AcrIIA4b with dCas9. (**F**) Top (i, iv) and front (ii, v) views of alphaFold model dCas9 in complexity with AcrIIA2 (left; homology PDB ID 6IF0) and AcrIIA4 (right; homology PDB ID 5VW1). (iii) Schematic diagram of the domain organization of SpyCas9. The RNA recognizing lobe (REC), two nuclease domains HNH and RuvC, and PAM-interacting domain (PAM-ID) of the dCas9 domains are colored according to the scheme in (iii). Contact maps of predicted interface residues of dCas9 with AcrIIA2 (**G**) and AcrIIA4a (**H**) within 8Å distance of interactions. The α-chains, β-sheets and loops (L1-5) of AcrIIA4a are labeled. Likewise, the IDRs and IOR are highlighted in orange. In panels (F)–(H), the probably optimal insertion sites are spotlighted in magenta.

To engineer a light-switchable system, we would like to identify the favorable sites for the insertion of the photoreceptor *A. sativa* LOV2 in AcrIIA4 and AcrIIA2 (the potent one) by analyzing the predicted structures of these Acr proteins (Fig. 2A). Structural analysis using AlphaFold3 revealed that although the two AcrIIA4 primary structures are 40% dissimilar, they possess nearly identical tertiary structures and hydrophobicity patterns (Fig. 2B and Supplementary Fig. S4A–C). However, the charge distribution of their residues (Supplementary Fig. S4D) and their electrostatic potentials on the surface differ slightly (Fig. 2C). More explicitly, although their surfaces are acidic, a common feature of the AcrIIA4 family [44], the F⍺ helix of AcrIIA4a is highly negative compared to that of AcrIIA4b. As for AcrIIA2, its structure has little resemblance to those of the AcrIIA4 family (Supplementary Figs S2B and S4A), because the molecular mechanisms by which these Acrs block DNA binding of Cas9 are different [38, 42].

As IDRs play key roles in protein engineering [45, 46], we computed the pLDDT scores—outlined by AlphaFold3, formerly by AlphaFold2 [32]—of AcrIIA2 along with AcrIIA4a and A4b. Due to the small size of Acr proteins, we defined IDRs as four amino acid sequences or above without 3D structures, having the pLDDT score <85, similar criteria reported previously [16]. Notably, with this computation of pLDDT scores, there are peptide sequences—at least 4 aa in length, with pLDDT <85 and 3D structures—that would not be otherwise classified as IDRs [16] and formerly proposed as 3D-folded IDRs [21]; we named IORs these peptide sequences. In other words, an IOR is four amino acid sequences or above with the pLDDT score <85, and natively possesses a 3D structure. With these defined IDRs and IORs, our analysis displays that AcrIIA2 is IDR-enriched (~30% of full sequence), 25%, 40%, and 20% among IDRs are around the loops L1 (21–29 aa), L3 (55–68 aa), and L5 (95–100 aa), respectively (Fig. 2D). Alternatively, AcrIIA4a and AcrIIA4b have short IDRs that extend in loops L3 (35–38 aa) and L5 (64–70 aa), alongside an IOR at their C-terminus, which is seven residues (84–90 aa) for A4a and four (84–87 aa) for A4b (Fig. 2E). The IDRs and IORs of these three Acr proteins contain a high proportion of acidic residues. This less-hydrophobic feature of IDRs is consistent with the pLDDT-score-defined IDRs in big-sized proteins [19–21], even though our selected Acrs are small-sized ones (<125 aa).

To determine the favorable LOV2-insertion sites—residues in a loop of Acr protein that are not in close contact with any dCas9 residue (distance from the dCas9 interfaces >8Å)—we generated alphaFold-predicted Acr–dCas9 complex models (Fig. 2F). To this end, we performed intercontact map analyses of these alphaFold models, in which AcrIIA2 or AcrIIA4 blocks the DNA binding pocket of dCas9, which possesses several domains (Fig. 2F). These analyses reveal that, as previously reported [38], AcrIIA2 interfaces with mainly the PAM-identification and RNA-recognizing lobe of dCas9 (Fig. 2G), whereas AcrIIA4a and A4b do so with every domain of the latter (Fig. 2H and Supplementary Fig. S4E)—explaining their robust inhibition of dCas9_fly (Fig. 1). Interestingly, IDRs of AcrIIA4a and A4b more strongly interact with dCas9 than their IORs, indicating that IDRs are functionally important for the interactions and not suitable for opsin LOV2 insertion (Fig. 2H and Supplementary Fig. S4E). Inferring from these Acr–dCas9 predicted interface regions, the residues 100–101 (Loop L5, adjacent to IDR5, distance from dCas9 interface >8Å) of AcrIIA2 and 62–63 (loop L5, adjacent to IDR2, distance from dCas9 interface >8Å) of AcrIIA4a along with A4b could be the optimum sites for LOV2 insertion (Fig. 2G and H, and Supplementary Fig. S4E). On that account, our most likely optimal insertion sites of LOV2 might not support the previous findings, proving that IDRs are the optimum sites of insertion of an opsin [9, 26]. Taken together, for these *in silico* analyses, not only did we predict the favorable sites for LOV2 insertion in AcrIIA2 and AcrIIA4, which are rather adjacent to IDRs, but we also proposed the existence of IOR in these small-sized proteins.

### A4b.LOV_63, a prototype photoswitchable component functional *in vivo*

**Figure 3.**
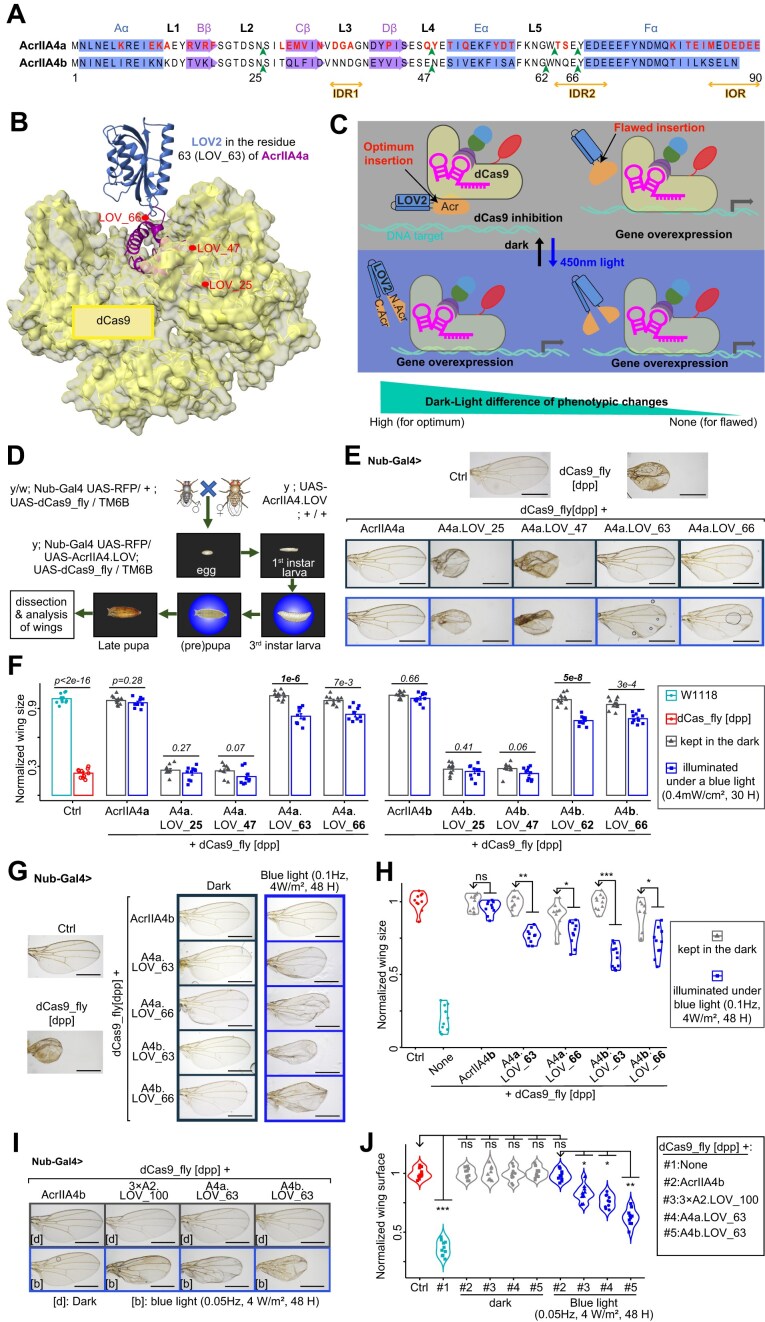
Engineered Acr proteins and their light-switchable to modulate gene overexpression *in vivo*. (**A**) Representations of secondary structure of AcrIIA4a and aligned with that of AcrIIA4b, and their selected LOV2 insertion sites (in green arrowhead). The amino acid sequences of alpha-chains, beta-sheets, and loops are colored in blue, medium orchid, and black, respectively. The IDRs and IOR are marked in orange. The red characters are the residues that differentiate AcrIIA4a from A4b. (**B**) Predicted structural model of the system for the LOV2 (dark blue) inserted in W63 of AcrIIA4a. The other LOV2 insertion sites are also highlighted in red. The surface of dCas9 is colored in semi-transparent yellow. (**C**) Rationale behind the optimum site of LOV2 insertion for inhibition of dCas9_fly. When LOV2 (dodger blue) is inserted in the optimum position of Acr protein (orange), the latter can inhibit dCas9 in the dark but is split by the active LOV2 under blue light, allowing dCas9_fly-mediated EnGup. (**D**) Experimental workflow. The crossing and their progenies were entirely kept in the dark, temporarily illuminated under a blue LED-light at the 3rd instar larval, prepupal, or pupal stage, and then put back in the dark before the dissection of the wings of adults. (**E**) Illustrative images of wings from flies co-expressing dCas9_fly-mediated *dpp* EnGup and LOV2-inserted AcrIIA4 at different positions. The blue-framed images were from the larvae exposed to a blue LED-light. (**F**) Data summary of normalized wing sizes described in panel (E). The numbers on the top of each pair of bars are the *P*-values of ANOVA tests of the dark–light difference with *n* = 10 female flies. (**G**) Representative images showing the light-responsiveness of LOV_63 of A4a and A4b compared to the positive control LOV_66. (**H**) Data summary of normalized wing sizes described in panel (G). It displays that LOV_63 of A4a and A4b is more light-responsive than LOV_66 for *in vivo* drosophila. Representative images (**I**) and summarized data (**J**) illustrate the light-sensitivity of AcrIIA4b compared with AcrIIA4a and triple dose AcrIIA2. In panels (E), (G), and (I), scale bars are 500 μm. The dark gray- and blue-framed images are the wings from flies entirely kept in the dark and temporarily illuminated with a blue LED light, respectively. In panels (H) and (J), *P*-values were calculated by ANOVA-test; *n* = 10 female flies; *ns*, *, ** and *** are, respectively, not significant, <0.01, <1e−3, and <1e−5 between the normalized phenotypes of the arrowhead-pointed groups and those of others. In E-J, the “Ctrl” is a dCas9_fly line without Gal4 driver.

Based on the abovementioned prediction of LOV2 insertion site, we built UAS plasmids expressing the prototypes of Acr–LOV2 fusion proteins and injected them into flies in order to validate photoswitchable endogenous gene regulation *in Drosophila*. More notably, LOV2—whose gap between C-terminal Jα helix, which unfolds upon photoactivation, and N-terminal is ~10 Å in the dark [9, 47] (Supplementary Fig. S5A)—was inserted into four sites of A4a and A4b: N25, Q47 (of A4a) and E47 (of A4b), G62 (of A4b) and W63 (of A4a), and E66 (Fig. 3A and B, and Supplementary Fig. S5B and C). The N25 (in Loop2) and Q47/E47 (in Loop4) close to the AcrIIA4–dCas9 interface act as negative controls, although the position 47 has been reported as a key residue of AcrIIA4–LOV2 engineering [27]; but the E66, the optimal insertion site in the previous human cell experiments [26], was included as a positive control (Fig. 3A and B, and Supplementary  S5B and C). In this case, the optimal site amongst these positions was picked out by the ability of Acr–LOV2, when coexpressed with dCas9_fly, to procure a maximum dark–light difference of phenotypes: the intact Acr protein that completely blocks dCas9-mediated changes of phenotypes is split by LOV2 under blue light, permitting endogenous gene activation (Fig. 3C). Before the dissections of the wings from adult flies for phenotypic analyses, all breeding processes were carried out in the dark; otherwise, pupae or larvae were illuminated under a blue light pulse (Fig. 3D).

After checking the wing phenotypes of adult flies whose larvae were kept in dark or exposed to blue light, we expectedly found that LOV_25 and LOV_47 of A4a and A4b, whether in dark or light conditions, exhibited phenotypes similar to dCas9_fly alone (Fig. 3E and F), suggesting that these two LOV2 insertions in A4a and A4b disrupted the inhibitory function of A4 proteins *in vivo*. The LOV2 insertions in W63 of A4a and in G62 of A4b, together with that in E66 of both AcrIIA4, were light-responsive, because wings were significantly smaller when larvae were exposed to a blue light (0.1 Hz, 0.4 mW/cm^2^) than when kept in the dark (Fig. 3E and F). Assessing the two adjacent positions (G62 and W63) in A4b, LOV_63 performed significantly better than LOV_62, as the G62 site is one residue apart from the IDR2 (Supplementary Fig. S6A and B). Different from LOV_25 and LOV_47 insertions disrupting inhibitory function of A4 (Fig. 3E and F), LOV_62 and LOV_63 insertions in A4b had no significant effect on wing size in dark conditions compared to that of A4b alone, indicating that the insertion of LOV2 in either G62 or W63 kept A4b protein functionally intact (Supplementary Fig. S6A and B). The in-depth examination of the insertions in W63 and E66 of A4a and A4b exhibited that A4b.LOV_66 defectively inhibited dCas9 in the dark, leading to less-consistent wing sizes, and A4b.LOV_63 produced a maximum dark–light phenotypic difference (Fig. 3G and H).

Based on our prediction (Fig. 2G), we also generated fly lines expressing A2.LOV_100, a potentially light-switchable fusion, and A2.LOV_45 (T45 in Cß; Supplementary Fig. S4B), a negative control (Supplementary Fig. S7A). Using genetic manipulation, we doubled the expression of AcrIIA2 (2× A2) (Supplementary Fig. S7B). As expected, 2× A2.LOV_45 disrupted the inhibitory effect of AcrIIA2; however, fruit flies coexpressing dCas9_fly along with 2× A2.LOV_100 had significantly smaller wing sizes under a blue condition (0.1 Hz, 4 W/m^2^) compared to those in the dark condition (Supplementary Fig. S7C and D). To further dose A2.LOV_100, we generated a fly line expressing tricistronic A2.LOV_100 (3×), which was able to more efficiently inhibit dCas9_fly than 2× A2.LOV_100 in dark condition (Supplementary Fig. S7E–G). Even so, the dark–light differences of 3× and 2× A2.LOV_100 was similar, meaning that the photoswitchability of Acr–LOV2 fusion did not depend on the dosage of AcrIIA2 (Supplementary Fig. S7G). Subsequent comparison of 3× A2.LOV_100 with LOV_63 of the two A4 variants displayed that these three variants were able to effectively inhibit dCas9_fly in the dark, and were light-responsive (Fig. 3I and J). A closer look, however, showed that A4b.LOV_63 among these three variants was more responsive to light (Fig. 3I and J). In short, although the light-sensitivity needed to be further improved, A4b.LOV_63 was a prototype for photoswitchable EnGup with dCas9_fly *in vivo*.

### Refining the length of IDR and IOR of A4b.LOV_63 increases its photoswitchability *in vivo*

**Figure 4.**
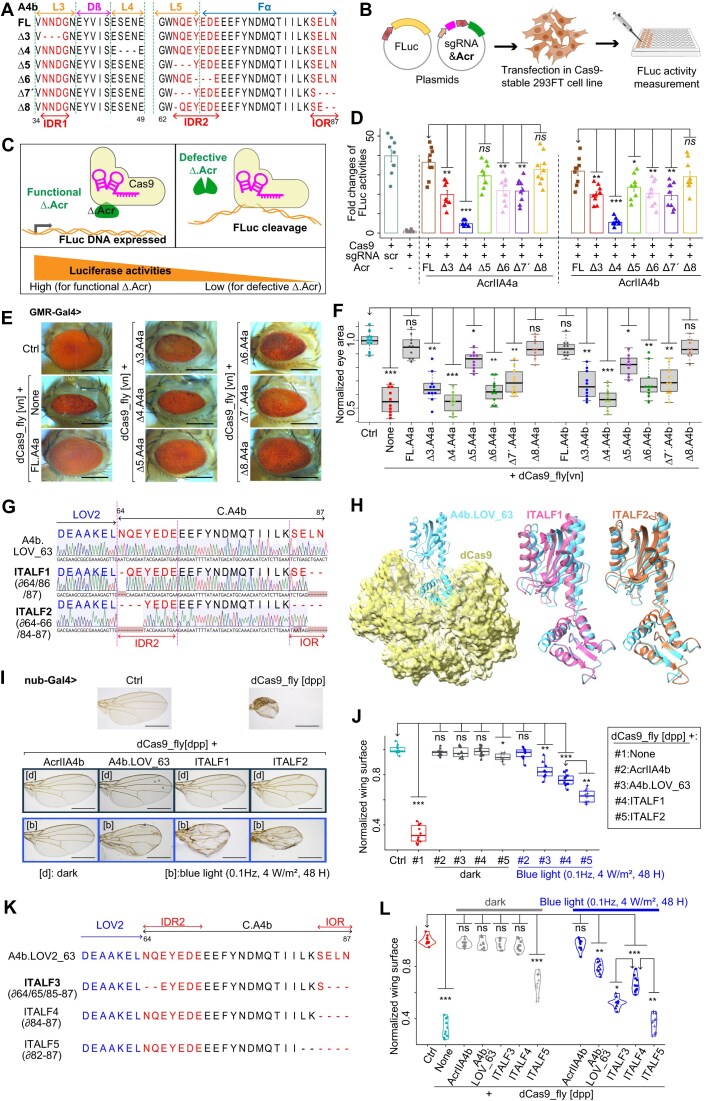
Optimization of A4b.LOV_63 photoswitchability *in vivo* by truncation of IDR2 and IOR. (**A**) Partial amino acid sequences of truncated AcrIIA4b variants aligned with the full-length one (FL). ∆7′ indicates a double truncation, and ∆8 is the deletion of tree residues in AcrIIA4b (∂64/86/87). The secondary structures are outlined. (**B**) Experimental design for testing the inhibitory efficiency of truncated Acrs. Cells stably expressing Cas9 were co-transfected with each truncated Acr, sgRNA targeting Luciferase, and a reporter Firefly Luciferase. (**C**) Rationale behind the identification of truncatable residues of Acr: high Luciferase activity indicates the truncated residues are not necessary for the Cas9-inhibiting functionality of the Acr variant, and vice versa. (**D**) Plot showing the results of Firefly Luciferase activities targeted by Cas9 according to the inhibition efficiency of co-transfected Acr. The AcrIIA4a&b are truncated corresponding to panel (A) and Supplementary Fig. S7A and B; “scr” means scrambled sgRNA. Bars represent mean values, error bars the standard deviation, and dots individual data points from *n* = 9. (**E**) Representative images of compound eyes from *Drosophila* co-expressing dCas9_fly and FL or truncated AcrIIA4a&b proteins mentioned in panel (A) and Supplementary Fig. S7A and B. (**F**) Quantitative data of eye surface area from dCas9_fly drosophila expressing truncated AcrIIA4A&b. (**G**) Partial sequence graphs of IOR/IDR-truncated Acr–LOV2 complexes ITALF1 and ITALF2 aligned with A4b.LOV_63, featuring their truncated residues ∂64/86/87 and ∂64–66/84–87, respectively. The sequence graphs were aligned with Benchling platform; the dashes or codons in blossom color are deletions or mismatches. (**H**) Generated alphaFold of ITALF1 (magenta) and ITALF2 (coral), aligned with A4b.LOV_63 (cyan), which interacts with dCas9 (yellow). Representative images (**I**) and statistical data (**J**) of wing phenotypes from dCas9_fly flies expressing Acr–LOV2 complexes in panels (G) and (H), and kept in the dark or exposed to blue-light pulse at the pupal stage. (**K**) Partial sequence graphs and corresponding peptide sequences of ITALF3, ITALF4, and ITALF5, whose residues 64–65/85–87, 84–87, and 82–87, respectively, are truncated, and aligned with A4b.LOV_63. (**L**) Statistical data set of normalized wing sizes from dCas9_fly drosophila expressing Acr–LOV2 complexes in panel (K), displaying the high and marginal photoswitchabilities of ITALF3 and ITALF4, respectively, and disrupted functionality of ITALF5. The scale bars in panels (E) and (I) are, respectively, 250 and 500 μm. In panels (G) and (K), the residues in blue and red are LOV2 and IDR2 plus IOR of C.AcrIIA4b. The dash (-) represents a single residue deletion. In panels (D), (F), (J), and (L), the “Ctrl” is a dCas9_fly line without Gal4. Also, the *P*-values * <.01, ** <1e−3, *** <1e−5 were calculated by one-way analysis of variance (ANOVA) of the differences between the normalized and quantified Luciferase activities [for panel (D)] or phenotypes [for panels (F), (J), and (L); with *n* = 10 female flies] of the arrowhead-pointed groups and those of others. In all panels, ∂ (lower case “Delta”) and ∆ (upper case “Delta”) refer to single and more than one residue deletions, respectively.

Since A4a has a longer IOR than A4b and the insertion of LOV2 in AcrIIA2 (L100) is in the IDR5, we reasoned that residues in IDR and IOR of A4 proteins would provide an opportunity to achieve an optimization of the light-sensitivity of our prototype A4b.LOV_63 *in vivo*. Realizing that IDR of AcrIIA5 was previously reported to be crucial for Cas9 inhibition [48], we created triple-residue-deletion variants (Fig. 4A and Supplementary Fig. S8A and B): ∆3, ∆4 as a negative control, ∆5, ∆6, double truncated ∆7′, and partially double truncated ∆8 (∂T64/E89/E90 for A4a and ∂N64/L86/N87 for A4b), in order to identify truncatable residues in IDRs and IOR of A4.

To assess their functionality, we co-transfected these truncated mutants, at first, with Cas9 plus sgRNA along with *Firefly Luciferase*, the sgRNA-targeted gene, in 293FT cells (Fig. 4B). In this case, Luciferase activity served as a reporter for *Luciferase* DNA cleavage by Cas9 when at low activity level, and for Cas9 inhibition by an A4 mutant when at high level (Fig. 4C). Luciferase assays revealed that all deletion mutants were relatively inefficient at blocking Cas9, except for two variants: ∆4 was completely dysfunctional, and ∆8 effectively inhibited Cas9. Explicitly, the inhibitory efficiency of ∆8 was comparable to that of FL A4a or A4b (Fig. 4D). These data were further confirmed by inhibition of dCas9 stably expressed in the 293FT cell line, which was co-transfected with sgRNA, truncated A4b, and the EGFP-targeted gene for transcriptional activation (Supplementary Fig. S8C). In these confirmational experiments, the proportion of EGFP-expressing cells is a reporter of dCas9 inhibition; it is high when an Acr mutant does not inhibit dCas9, and vice versa (Supplementary Fig. S8D). In contrast to ∆4, the results showed that the ∆8 mutant efficiently blocked dCas9 activities, similar to FL.A4b (Supplementary Fig. S8E and F). To determine the truncatable residues *in vivo*, we inserted these IDR/IOR-truncated mutants at *attp40*. When they were coexpressed with dCas9_fly, the ∆8 mutants of A4a and A4b were able to rescue the dCas9_fly-mediated phenotypes (Fig. 4E and F). In addition, all other mutants were relatively inefficient in inhibiting dCas9 *in vivo*, except ∆4 (Fig. 4F). These results demonstrate that certain residues in IDR2 and IOR can be truncated without disrupting A4 function *in vivo*.

Knowing the truncatable residues in IDR2 and IOR of A4 and the light-response of the prototype A4b.LOV_63 in endogenous gene regulation *in vivo* (Fig. 3I and J), we generated flies expressing “IOR/IDR-Truncated AcrAIIA4-LOV2 Fusion (ITALF)” variants: ITALF1 (∂N64/L86/N87), a partially IDR- and IOR-truncated A4b, and ITALF2 (∂N64-E66/S84-N87), a fully IOR- and partially IDR-truncated A4b (Fig. 4G). Alignments of generated alphaFold models of these variants showcase that they are structurally similar to A4b.LOV_63; thus, they occupy the same position in dCas9 as the latter (Fig. 4H). Comparison of phenotypes showed that ITALF2 did not stably block dCas9_Fly in dark *in vivo* (Fig. 4I). In terms of light-sensitivity, although the two ITALFs were more light-responsive than A4b.LOV_63, ITALF2 was significantly more sensitive to blue light than ITALF1 (Fig. 4J).

Further, we generated additional A4b-LOV2 variants: ITALF3 with truncations of two IDR2 (∂N64/Q65) and three IOR residues (∂E85-N87), ITALF4 with an entirely truncated IOR, and ITALF5 with a truncation IOR and beyond (∂L82-N87) (Fig. 4K). Notably, strong light-response was yielded with ITALF3; truncation of the entire IOR (in ITALF4) led to efficient dCas9_fly inhibition in dark and fair, but not optimal, photoswitchability; truncation beyond the computed IOR (in ITALF5) abrogated the inhibitory activity of dCas9_fly in dark (Fig. 4L and Supplementary Fig. S9A). Similarly, IOR truncation of A4a.LOV_63 (ITALF6 and ITALF7) also effectuated reliable dCas9 inhibition in dark and moderate response under blue light (Supplementary Fig. S9B–D). Moreover, the mutation of N64/Q65/E85/L86/N87 of A4b to glycine (G) marginally had effects on phenotypes, while ITALF3 significantly displayed reduced phenotypes under blue light (Supplementary Fig. S9E–G). These findings demonstrate that fine-tuning of IOR and IDR length significantly enhances the light-sensitivity of the A4b.LOV_63 prototype *in vivo*.

### Optimization of LOV2 and light properties enables rapid photoactivation of ITALF

**Figure 5.**
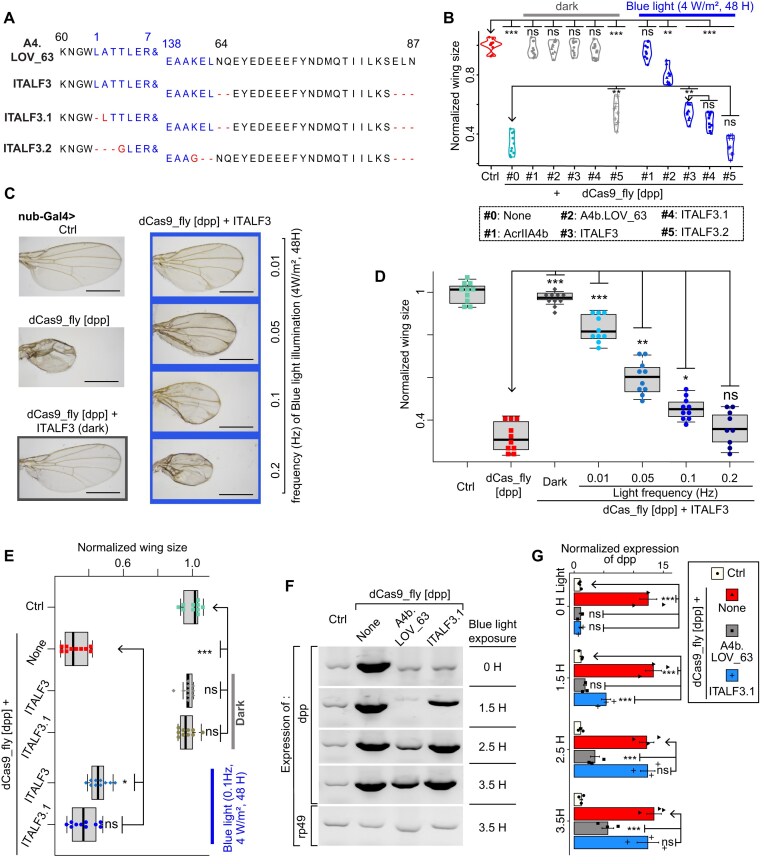
Mutation of LOV2 and adjustment of light properties of ITALF3. (**A**) Peptide sequences of ITALF3, ITALF3.1 and ITALF3.2, whose LOV2 (in blue) of which are truncated, and aligned with A4b.LOV_63 and ITALF3. The ampersand signs (&) are 8–137 residues of LOV2. The dash and letter in red represent truncated and mutated residues. (**B**) Plot of normalized wing sizes from dCas9_fly drosophila expressing Acr–LOV2 complexes in panel (A). It shows that ITALF3.1 is marginally light-sensitive than ITALF3, and ITALF3.2 is dysfunctional. The larvae of these flies were kept in the dark or irradiated with blue light (~0.08 Hz, 4 mW/m^2^ for 48 h). Representative images (**C**) and statistical data (**D**) of comparison of wing phenotypes from ITALF3 exposed at different light frequency at a larval stage. The wings were from fly adults, the larvae of which were kept in dark or irradiated with blue light (4 mW/m^2^ for 48 h). (**E**) Comparison of ITALF3 and ITALF3.1: statistics of wing phenotypes from dCas9_fly drosophila, expressing ITALF3 or ITALF3.1, and the larvae of which were exposed to blue light (0.1 Hz, 4 W/m^2^, 48 h). Gel images (**F**) and quantitative data (**G**) of RT-PCR results showing the light-sensitivity of ITALF3.1 compared to A4b.LOV_63. The RNAs were extracted from early hatched fly wings right after light exposure. The expression of *rp49* represents a loading control. In panels (B), (D), (E), and (G), the labels *ns*, *, **, *** are *P*-values not significant, <0.01, <1e−3, and <1e−5, respectively, that were calculated by ANOVA-test of the difference between the normalized wing sizes or eye surface area of the arrowhead-pointed groups and those of others. In panels (B)–(G), the “Ctrl” is a dCas9_fly line without Gal4.

To further improve the light-sensitivity of ITALF, we explored truncation of LOV2 termini, a modification known to enhance photoswitchability [49, 50]. Established on that, we designed ITALF3.1 and ITALF3.2 upgraded versions with truncated LOV2 (∂A2) and the reported short-LOV2 (∂L1-T4/K141-L143) [49, 50], respectively (Fig. 5A). When coexpressed with dCas9_fly, ITALF3.1 exhibited marginal advantages compared to ITALF3; ITALF3.2 hardly inhibited dCas9 in the dark (Fig. 5B). Although the short-LOV2 (ITALF3.2) was used to control nanobodies and transcription factors [49, 50], it does not suitable for AcrIIA4 (Fig. 5B). Our data suggest that the hydrophobic leucine residues at the LOV2 termini are critical for maintaining a fully closed LOV2 dark state.

To enhance the efficiency of our system for photoswitchable EnGup *in Drosophila*, we also refined light exposure conditions. Compared to 24 and 72 h, 48 h of light exposure induced a reasonable phenotype and mitigated phototoxicity (Supplementary Fig. S10A and B). Noting that the low irradiances caused no significant effect on the wings, while the high one (10 W/m^2^) was toxic, 4 W/m^2^ irradiance—which is almost as bright as for *in vitro* experiments [11, 12]—showed reasonable phototoxicity and light-response in the context of ITALF3 (Supplementary Fig. S10C and D). Next, by calibrating the light pulse rate, we observed a limited degree of phenotypic changes for the low frequency (~0.01 Hz), which is sufficient to maintain the adduct state of *A. sativa* LOV2 that we used [51] (Fig. 5C and D), unlike other LOV variants [52, 53]. Besides, we observed phenotypic changes of dCas9_fly-ITALF3 and of dCas9_fly-ITALF3.1 comparable to those of dCas9_fly alone for the pulse of 0.2 and 0.1 Hz (4 W/m^2^ and 48 h), respectively (Fig. 5D and E, and Supplementary Fig. S10E). These indicated that an optimized condition has been achieved for EnGup *in Drosophila* with ITALF3 and ITALF3.1, the slightly better one.

Next, we evaluated properties of our module. An examination at the mRNA level showed that the maximal upregulation can be reached after 2.5 h of blue light exposure for ITALF3.1, while over 3.5 h for A4b.LOV_63 (Fig. 5F and G). Correspondingly, we implemented dark-recovery experiments with these Acr–LOV2 variants: flies were kept in the dark after their exposure to blue light. The quantification of mRNA level displayed that ITALF3.1 reached the full recovery after 3.5 h of incubation in the dark, while A4b.LOV_63 still needed more time (Supplementary Fig. S11A and B). Additionally, we checked the toxicity *in vivo* of ITALF3.1 by expressing it in adipose tissues and found normal survival rates (Supplementary Fig. S11C). We also carried out experiments in human cells to compare our module with split-dCas9 [54], which can reassemble and be functional under blue light only (Supplementary Fig. S11D). Our results showed that ITALF3.1 retained its light-sensitivity and minimal leakage in the dark compared to split-dCas9 and A4b.LOV_63 (Supplementary Fig. S11E and F). Together, ITALF3.1 achieves an optimally light-sensitive and rapid EnGup *in vivo*.

### ITALF enables the investigation of the biological functions of genes in different tissues *in vivo*

**Figure 6.**
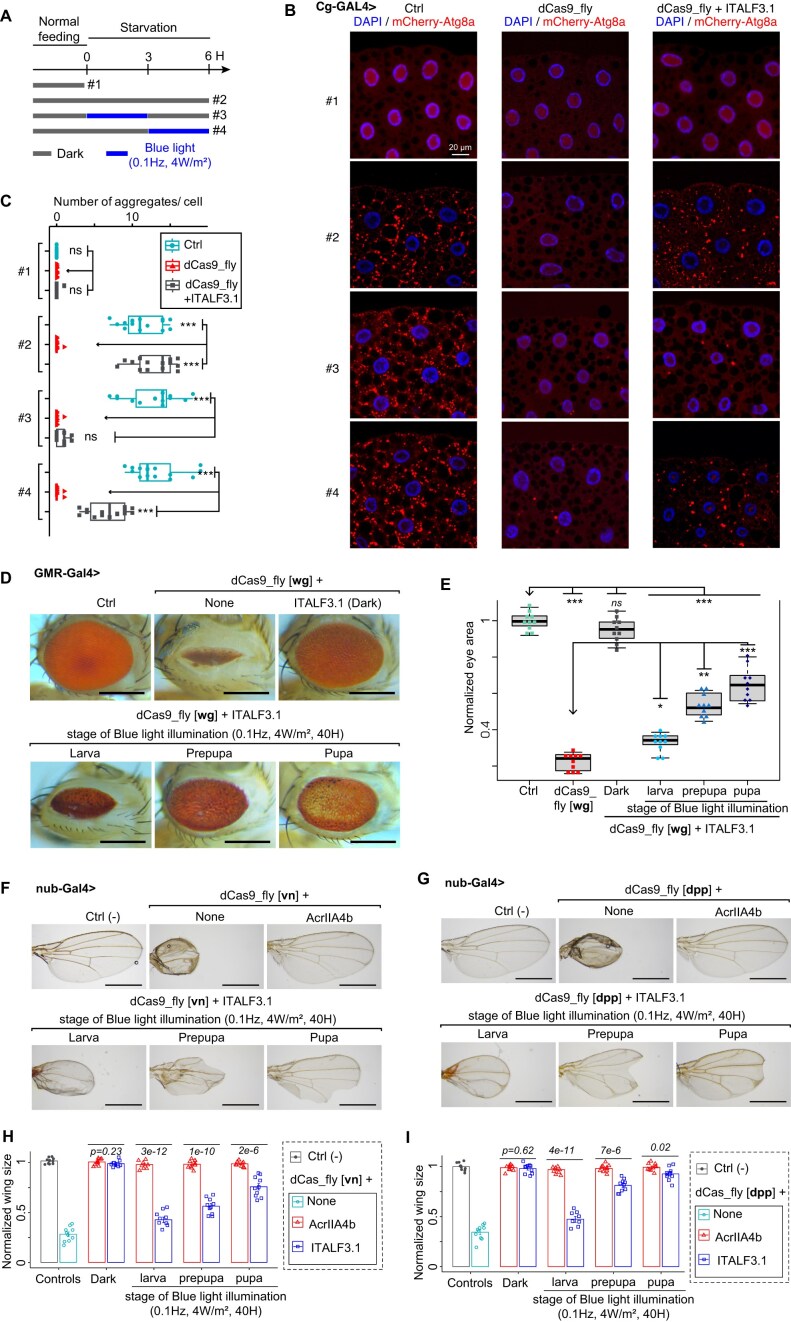
Application of ITALF on targeting different tissues and genes *in vivo*. (**A**) Experimental paradigm for investigating ITALF3.1-mediated *Wnt4* EnGup through expression of mCherry–Atg8a (in red). Larvae were normally fed or starved either in the dark (dark gray lines) or under a blue light (blue lines), before mounting their adipose tissues. (**B**) Confocal images of L3 fat body adipocytes showing localization and expression of mCherry–Atg8a in red (driven by Cg-GAL4). Nuclei stained with DAPI (blue). (**C**) Plot quantifying mCherry–Atg8a puncta per cell from data in panel (B). (**D**) Image samples illustrate the eye phenotypes of dCas9_fly drosophila adults expressing ITALF3.1. These flies, whose larvae, prepupae or pupae were illuminated under a blue light pulse (0.1 Hz, 4 mW/m^2^ for 40 h), expressed *wg* EnGup. The scale bars are 250 μm. (**E**) Plot summarizes the data in panel (D). Representative images of wing phenotypes from dCas9_fly flies, expressing ITALF3.1 or AcrIIA4b and dCas9_fly targeting *vn* gene in panel (**F**) or *dpp* in panel (**G**). The larvae, prepupa, or pupae of these flies were irradiated with blue light (0.1 Hz, 0.4 mW/cm^2^) for 40 h. The scale bars are 500 μm. Statistics from panel (F) in panel (**H**) and panel (G) in panel (**I**). *P-*values calculated by ANOVA-test are the mean of normalized wing sizes from dCas9_fly flies expressing ITALF3.1 compared to those expressing AcrIIA4b. In panels (C) and (E), the labels *ns*, *, **, *** are *P*-values not significant, <0.01, <1e−3, and <1e−5, respectively, which were calculated by ANOVA-test of the difference between the mCherry–Atg8a puncta per cell (for panel C) and normalized eye area (for panel E) of the arrowhead-pointed groups and those of others. In panels (D)–(I), the “Ctrl” is a dCas9_fly line without Gal4.

We next sought to demonstrate the application of our light-switchable gene regulation module (ITALF3.1) in optogenetically controlling gene expression of other tissues *in vivo*. We started with investigating whether, in adipose tissues, ITALF3.1 can control other EnGup, such as of *Wnt4* gene, which has been speculated to be involved in autophagy [55]. For this investigation of ITALF3.1 in adipose tissues, we coexpressed ITALF3.1, *Wnt4* EnGup and mCherry-marked autophagy-related gene 8a (Atg8a) using Cg-Gal4 driver. Then, before mounting their adipose tissues on slides, larvae were subjected to four types of experimental paradigms: #1 fed normally; #2 starved for 6 h in the dark; #3 starved for 6 h, during the first half (3 H) of which the larvae were exposed to blue light; and #4 starved for 6 h, during the last half of which the larvae were exposed to blue light (Fig. 6A). Atg8a was expressed in the nucleus under normal conditions, and formed puncta in the cytoplasm when larvae were under stress or starved (Fig. 6B), as previously reported [56]. But, the *Wnt4* EnGup suppressed the formation of these Atg8a puncta, meaning that our results provide insights into the involvement of *Wnt4* in autophagy that was previously speculated [55]. More intriguingly, the coexpression of *Wnt4* EnGup and ITALF3.1 under food stress allowed Atg8a-puncta formation in the experimental paradigms #2 (in the dark) and #4, and suppression of this formation in #3 (Fig. 6B and C). Thus, our system enables to optogenetically control of biological processes in a fine time window *in vivo*.

To confirm functionality of our system in other tissues, we generated a fly line for wingless (*wg*) EnGup, leading to robustly smaller eyes—compared with *dpp* and *vn* EnGups—when driven by GMR-Gal4 and also smaller wings when driven by nub-Gal4 (Supplementary Fig. S12A–D). Of note, we evaluated the on- and off-target effects in the generated line and detected no changes in the mRNA level of the off-target genes (Supplementary Fig. S12E and F). Capitalizing on these results, we temporarily upregulated *wg* expression in eyes by coexpression of dCas9_fly and ITALF3.1 at three different stages of development. Light-irradiated larvae gave rise to adults with a narrower eye area, whereas the pupae to flies with a facetless eye area (Fig. 6D). The dark–light difference of eye phenotypes was significant at every stage, although that at the larval stage was most significant (Fig. 6E). Using our light-switchable gene regulation module, our results showed the ability of the *wg* gene to disrupt photoreceptor formation when expressed during *Drosophila* early developmental stages (Supplementary Fig. S12G), similar to what was previously shown by exogenous gene overexpression [57, 58]. These, therefore, validate that our light-switchable module can be used for different tissues and at different stages of development.

To generalize this approach, we also used our light-switchable system to regulate *vn, dpp*, and *wg* expression in wings. When *vn* EnGup was activated by light at larval and prepupal or pupal stages of development, we observed, respectively, proximally distorted and distally deformed wings in fly adults (Fig. 6F). Light-activation of *dpp* at larval and prepupal or pupal stages caused, respectively, proximally distorted and distally notched wings (Fig. 6G). Light-activation of *wg*, finally, marginally reduced wing size when expression was turned on at larval and prepupal but not pupal stage (Supplementary Fig. S12H and I). The EnGups of *vn* and *dpp* both significantly reduced adult wing size (Fig. 6H and I), in agreement with previous studies using exogenous gene overexpression [59–62]. In summary, our results prove the efficiency of our system to optogenetically regulate endogenous gene expression in a reasonable time manner.

## Discussion

This study represents the first report of AcrIIA4 and AcrIIA2 application in *Drosophila*. While AcrIIA4 is well used in animals [28, 63], the AcrIIA2 application *in vivo* has been restricted to yeast [64]. In *Drosophila*, an inhibitor or antagonist is needed to defend against the toxicity of continuous CRISPR–Cas activity, which is lethal when expressed in vital tissues [7]. Besides preventing CRISPR from off-target effects [65] and cellular toxicity [66, 67], Acr proteins have been demonstrated to maintain on-target editing while reducing off-target editing in human cells [44], to generate “write-protected” cells that prevent future gene editing as well as to control CRISPR-based gene regulation circuits [68]. This work reports for the first time that wild-type AcrIIA2, when sufficiently titrated *in vivo* at room temperature, efficiently inhibits Cas9 (Fig. 3H and I, and Supplementary Fig. S6), thus expanding the functional repertoire of Acr proteins.

Here, we demonstrate the application of our system to phenotypic modelling and autophagy investigation, but users can combine this system with Cas9, CRISPRi [8] and dCas9_fly [7]—through already available stocks—to mediate light-dependent editing and expression of any endogenous gene. This capability allows for *in vivo* cellular reprogramming and disease modeling under light control. Moreover, the versatility of ITALF in targeting genome-wide genes complements the presently available optogenetic approaches designed for transgenes [4–6]. Conceptually, even though LOV2-inserted Acr proteins have been used in cell cultures [25, 26], *E. coli* [27] and insects [28, 63], the current work is the first to develop such a system in *Drosophila*. Notably, the success of prototype A4b.LOV_63 was not optimally predictable from previous findings, carried out *in vitro* cell cultures, which emphasizes that the insertion of LOV2 around the IDR E66 or Y67 is preferable for AcrIIA4 [26]. These inconsistent results from cell cultures and animal experiments could be due to: (i) the residue mismatches of AcrIIA4 we used; (ii) or most plausibly, the strong binding specificity of IDRs of AcrIIA4 in inhibiting Cas9 *in vivo*. Such binding specificity of IDRs, which instigates more in-depth investigation of Acr–Cas9 interactions, has been pointed out to determine the functionality of transcriptional factors in yeast and mouse [10–15].

We optimized our prototype by refining the length of IDR and IOR of AcrIIA4 (Fig. 4 and Supplementary Fig. S10). While IDR is a widely accepted concept, its prediction in proteins remains an ongoing challenge, with computational methods continuously evolving [22, 69–72]. The generality of IDRs in functional interactions is still being explored [69], and their presence in small AFDB proteins has not been previously reported. Our approach provides another angle for understanding IDRs of small proteins. Regardless of the size of Acr proteins, our data support the ability of IDRs to regulate the stability and function of proteins as reported in other studies in *Drosophila* [73–75]. On the other hand, IOR is a newly proposed concept [21]. The IOR of AcrIIA4a is more acidic and marginally longer IOR than that of AcrIIA4b (Fig. 2C and E), a reason why A4b.LOV_63 is more light-responsive compared to the other prototypes (Fig. 3I and J). Likewise, IOR-truncated ITALF4 and ITALF6 along with ITALF7 are more light-sensitive than the A4b.LOV_63 and A4a.LOV_63, respectively (Fig. 4K and L, and Supplementary Fig. S9B–D). These imply that residues of IOR in AcrIIA4, and probably in small-sized proteins, can be mutable and truncatable for a particular purpose. Structurally, IOR of AcrIIA4 does not more strongly interface Cas9 than IDRs; considering this, it mechanistically contributes to Acr–CRISPR interaction like a kinetic stabilizer. Thus, IOR might arguably have important implications for the engineering of Acr and small-sized proteins.

In summary, this study pioneers the use of Acr proteins and their engineering for light-regulated endogenous gene expression in *Drosophila*. These prototypical optogenetic tools were optimized by truncating residues of IDR and IOR, which are structures herein characterized for the first time *in vivo* for small-sized Acr proteins. The broader applicability of IDR and IOR concepts in other small-sized proteins remains an open question for future research.

## Supplementary Material

gkag244_Supplemental_Files

## Data Availability

All data supporting the findings of this study are available within the paper and the supplementary information. All injected plasmids are deposited in BioStudies (accession number S-BSST2095), and all relevant fly lines are also available.
